# 

**Published:** 1906-06

**Authors:** 


### CONTENTS.

ORIGINAL COMMUNICATIONS—

MENINGITIS. By James B. Baird, M.D., Atlanta, Ga. .......... 145

AN UNUSUAL COMPLICATION FOLLOWING TYPHOID FEVER. REPORT OF CASE. By J. E. Sommerfield, M.D., Atlanta, Ga. .......... 161

SUGGESTION OR SUGGESTIVE THERAPEUTICS IN THE PRACTICE OF MEDICINE. By G. T. Kesner, M.D., Decatur, Ga. .......... 163

IS THERE A CONTINUED FEVER OTHER THAN TYPHOID? By J. W. Palmer, M.D., Ailey, Ga. .......... 170

ENTEROCOLITIS. By O. W. Cobb, M.D., Easthampton, Mass. .......... 176

CORRESPONDENCE .......... 178

EDITORIAL NOTES AND COMMENTS—

"SUBSIDIZED JOURNALS," "CANNED EDITORIALS" .......... 180

NEW STATE MEDICAL SOCIETY .......... 183

THE AUGUSTA MEETING OF THE MEDICAL ASSOCIATION OF GEORGIA .......... 184

NEWS AND NOTES .......... 186

CONTENTS CONTINUED .......... X

